# Improving diagnosis and management of pediatric ovarian masses: development of a risk stratification model incorporating sonographic and clinical features

**DOI:** 10.1186/s12887-025-06488-6

**Published:** 2026-01-22

**Authors:** Likai Chu, Zhiming Chen, Mingzhi Zhang, Tianna Cai, Min Zhang, Shuangquan Lu

**Affiliations:** 1 Department of Ultrasound, Children’s Hospital of Soochow University, Zhong Nan Road No.92, Suzhou, Jiangsu 215000 China; 2 Department of Radiology, Children’s Hospital of Soochow University, Suzhou, China; 3 Department of Pathology, Children’s Hospital of Soochow University, Suzhou, China

**Keywords:** Ovarian mass, Pediatric surgery, Ovarian malignancy, Sonography, Gynecology

## Abstract

**Objective:**

To develop and validate a pediatric-specific prediction model for discriminating malignant from benign ovarian masses in Chinese children, aiming to reduce unnecessary surgeries for physiological follicular cysts.

**Methods:**

This single-center retrospective study analyzed 344 consecutive patients ≤ 18 years undergoing ovarian surgery (2018–2024). Three blinded radiologists assessed sonographic parameters: maximum mass diameter and solid component proportion (Categorized as < 20%, 20–40%, 40–60%, 60–80%, > 80%). Multivariate logistic regression integrated clinical features, tumor markers, and sonographic variables to construct a malignancy prediction model. Diagnostic performance was evaluated by receiver operating characteristic (ROC) analysis.

**Results:**

Germ cell tumors (GCTs) predominated (72.7%, 253/348), with malignant lesions comprising 11.5% (40/348). Solid component proportion > 80% was the strongest malignancy predictor (odds ratio[OR] = 576.5, 95% confidence intervals [CI]: 74.0–4,492.6; *p* < 0.001). The combined model (Mass size + Solid component proportion) achieved superior diagnostic accuracy (Area under the curve [AUC] = 0.93, sensitivity 87.5%, specificity 83.2%), outperforming single parameters (Solid component proportion AUC = 0.86; Mass size AUC = 0.76). In addition to key clinical discriminators such as older age, absence of precocious puberty, and larger tumor size, the exclusive presence of sonographic features like septations (28.3%) and calcifications (5.7%) in epithelial tumors (*p* < 0.001 vs. follicular cysts) provides a reliable basis for differentiation, enabling a significant reduction in unnecessary surgeries for physiological cysts.

**Conclusion:**

This study establishes an evidence-based prediction model for Chinese pediatric ovarian masses, redefining malignancy risk stratification through quantitative sonographic thresholds. Furthermore, it identifies key discriminators (Septations, Calcifications, alongside Age, Precocious puberty and Mass size) to differentiate physiological follicular cysts from neoplastic epithelial tumors. The integration of solid component proportion > 40% and tumor biomarkers optimizes preoperative decision-making, which can significantly reduce unwarranted surgery for benign conditions while ensuring timely intervention for high-risk cases.

## Introducton

Ovarian masses represent the most prevalent gynecological neoplasms in children and adolescents, accounting for 1–2% of all pediatric malignancies. Strikingly distinct from adults—where epithelial carcinomas predominate—pediatric cases are dominated by GCTs (60–70%), predominantly benign mature teratomas, creating critical opportunities for fertility-preserving management [1, 2].

Early diagnosis of pediatric ovarian malignancies is well-established to significantly improve prognosis, even for advanced-stage tumors [3, 4]. However, progress is hindered by several persistent clinical dilemmas. First, adult prediction tools (e.g., The risk of ovarian malignancy algorithm [ROMA], Assessment of different neoplasias in the adneXa [ADNEX] models) are of limited use in children due to a fundamental mismatch: they target epithelial carcinomas and rely on CA-125 and HE4, whereas pediatric practice is dominated by GCTs, for which AFP and β-HCG are critical biomarkers [5, 6]. Moreover, although pediatric-specific risk models exist, they often rely on dichotomous imaging descriptors (e.g., solid vs. cystic) [7, 8]. However, since benign teratomas frequently contain solid elements like fat or bone [9], this simplistic dichotomization is inherently inaccurate for discriminating malignancy. Second, sonographic similarities between functional follicular cysts and surgically relevant epithelial tumors lead to unnecessary resections of physiological cysts, causing irreversible fertility damage. This clinical dilemma is under-represented in existing pediatric prediction models, which primarily focus on malignancy risk rather than also discriminating between types of benign lesions requiring different management. Third, current studies remain limited by small samples, while large-scale histopathological data from various populations remain scarce despite the potential for ethnic variations in tumor distribution. These gaps perpetuate reliance on subjective clinical judgment, leading to inconsistent surgical decision-making [10, 11].

To address these limitations, we conducted this large, single-center retrospective study of Chinese pediatric patients. The primary aims were: ① Systematically characterize the age-stratified pathological landscape of Chinese pediatric ovarian masses; ② To develop and validate a malignancy prediction model that integrates quantified sonographic parameters—specifically, the maximum diameter and a five-tiered categorical assessment of solid component proportion—with relevant clinical features and pediatric-appropriate tumor markers (AFP, β-HCG); ③ Establish objective clinical features and imaging discriminators for follicular cysts versus epithelial tumors.

This study introduces several key innovations to the field of pediatric ovarian mass management. First, we developed a novel, pediatric-specific predictive model that builds upon and refines existing tools [7]. Unlike adult-centric models or prior pediatric studies that often rely on dichotomous imaging descriptors, our model integrates pediatric-relevant tumor biomarkers (AFP, β-HCG) with quantitatively assessed sonographic parameters, most notably utilizing the solid component proportion as a graded categorical variable. Second, we established explicit and objective discriminators—specifically, the presence of septations or calcifications, alongside patient age and mass size—to differentiate physiological follicular cysts from neoplastic epithelial tumors, a common diagnostic pitfall leading to unnecessary surgery. By incorporating this novel, quantified imaging approach and directly addressing the follicular cyst dilemma, our model provides a significant advance over existing risk stratification tools. It is designed to optimize preoperative decision-making, with the dual goal of preserving fertility in low-risk children by avoiding unwarranted surgery while ensuring timely intervention for high-risk malignancies.

## Methods

### Participants

This single-center retrospective cohort study was approved by the Ethics Committee of Children’s Hospital of Soochow University (No. 2025CS184), with waiver of informed consent for anonymized retrospective data analysis. We included all consecutive patients ≤ 18 years who underwent ovarian surgery between January 2018 and December 2024. Exclusion criteria: ① Non-ovarian masses (e.g., Metastatic tumors to ovary); ② History of ovarian malignancy; ③ Incomplete clinical-sonographic data; ④ Disorders of sexual development. Finally, 344 patients were enrolled.

### Data collection

In this study, we collected various demographic, clinical, and imaging data of pediatric patients with ovarian masses, including age; clinical presentation such as abdominal pain and precocious puberty; laterality of the mass; and tumor markers including AFP, CA125, β-HCG, Carcinoembryonic Antigen(CEA) and estradiol. Sonographic parameters were systematically assessed, including maximum mass diameter and solid component proportion (categorized as < 20%, 20–40%, 40–60%, 60–80%, and > 80%), as well as the presence of septations and calcifications. Additionally, histopathological results were recorded for all surgically resected masses.

Three blinded pediatric radiologists (> 10 years’ experience) assessed maximum diameter (mm) and solid component proportion (5-tiered: < 20%, 20–40%, 40–60%, 60–80%, > 80%). Septations were defined as internal linear echoes ≥ 1 mm; calcifications as hyperechoic foci with acoustic shadowing.

### Statistical analysis

Normality of continuous variables was assessed by Shapiro-Wilk test. Normally distributed data are presented as mean ± SD (Independent t-test), non-normal as median Interquartile range [IQR] (Mann-Whitney U test). Categorical variables as frequency (%) (χ² or Fisher’s exact test). Binary logistic regression identified malignancy predictors with variable entry criterion: univariate *p* < 0.05. ROC analysis evaluated diagnostic performance. Analyses used SPSS 26.0 and Graphpad Prism 9.

## Results

**Fig. 1:**
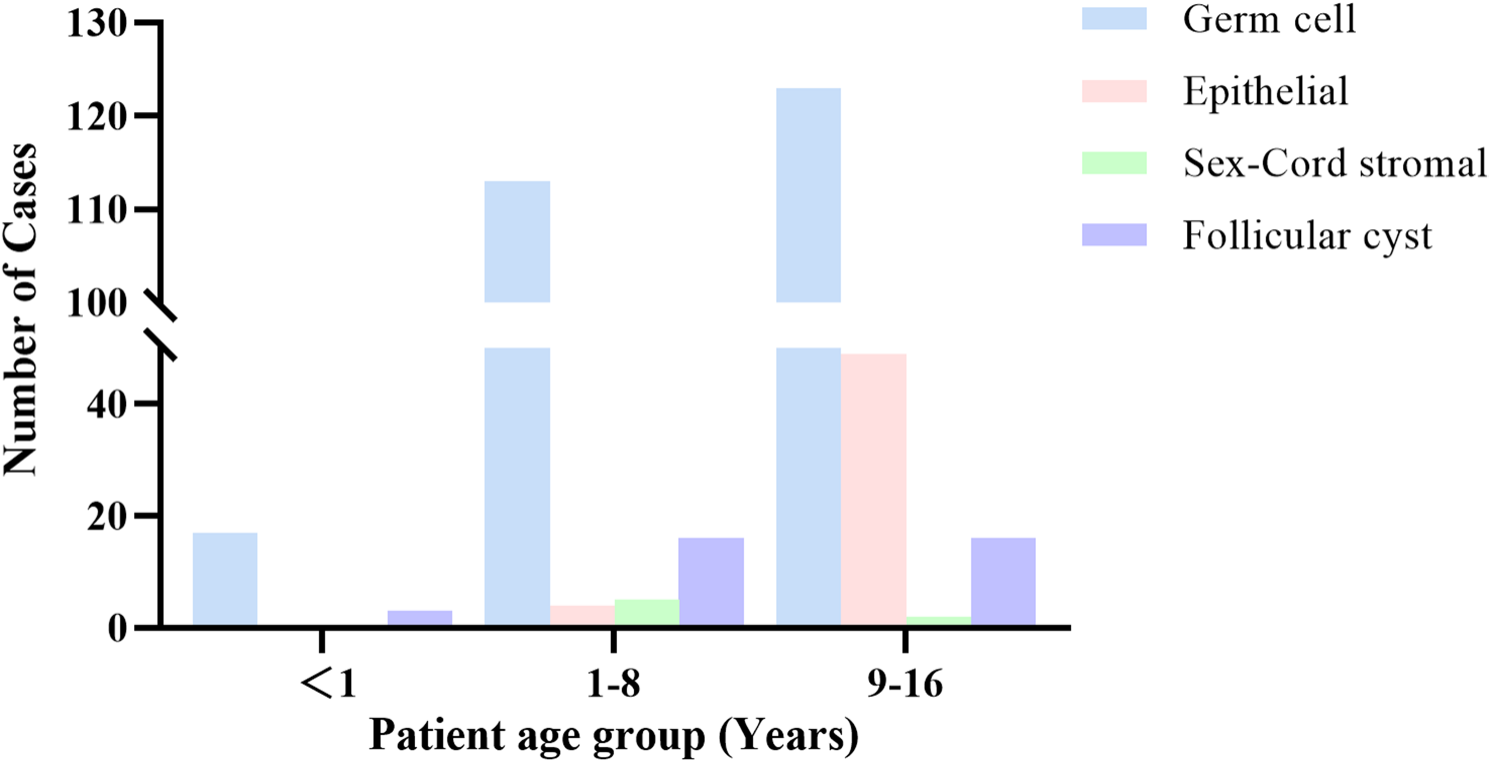
Distribution of ovarian mass pathologies across age groups (< 1, 1–8, 9–16 years)

Among 348 ovarian masses from 344 pediatric patients undergoing surgery (Including 4 patients with bilateral masses) (Table 1), GCTs constituted the predominant pathology (253 cases, 72.7%), followed by epithelial tumors (53 cases, 15.2%), sex cord-stromal tumors (7 cases, 2.0%), and follicular cysts (35 cases, 10.1%). Mature teratoma was the most common subtype (219/253, 86.6%), with immature teratoma (22/253, 8.7%), yolk sac tumor (4/253, 1.6%), dysgerminoma (4/253, 1.6%), and mixed germ cell tumors (4/253, 1.6%) comprising the remainder. Serous cystadenoma (28/53, 52.8%) and mucinous cystadenoma (25/53, 47.2%) were observed in Epithelial tumors. Sex cord-stromal tumors are included juvenile granulosa cell tumor (4/7, 57.1%), Sertoli-Leydig cell tumor (2/7, 28.6%), and fibroma (1/7, 14.3%). Age distribution analysis (Fig. 1) revealed epithelial tumors predominantly occurred in the 9-16-year age group (49/53, 92.5%), whereas GCTs and follicular cysts showed no significant age-specific predominance across groups.

**Table 1:** Histopathological classification of 348 pediatric ovarian masses by cell type and age group

Histological diagnosis of tumor	<1(y)	1–8(y)	9–16(y)	Total	Proportion ^a^
Germ cell	**17**	**113**	**123**	**253**	**72.7%**
Mature teratoma	16	93	110		
Immature teratoma	1	14	7		
Yolk sac	0	1	3		
Dysgerminoma	0	3	1		
Mixed germ cell tumors	0	2	2		
Epithelial	**0**	**4**	**49**	**53**	**15.2%**
Serous cystadenoma	0	2	26		
Mucinous cystadenoma	0	2	23		
Sex-cord stromal	**0**	**5**	**2**	**7**	**2.0%**
Juvenile granulosa cell	0	3	1		
Sertoli-Leydig	0	2	0		
Fibroma	0	0	1		
Follicular cyst	3	16	16	**35**	**10.1%**

^a^ Values are n (%) of 348 cases

After excluding follicular cysts (*n* = 35), 313 ovarian masses from 309 patients were classified by malignancy status, comprising 273 benign lesions (Including 4 bilateral cases) and 40 malignant lesions. Comparative analysis of clinical characteristics, laboratory markers, and ultrasound parameters is presented in Table 2. Malignant cases occurred at a significantly younger age than benign cases (7.4 ± 2.9 years vs. 8.8 ± 3.8 years; *p* = 0.01). No significant difference in laterality was observed (*p* = 0.07). Abdominal pain was the most common presenting symptom in both groups (Benign: 54.3%, Malignant: 52.5%; *p* = 0.834), while precocious puberty was significantly more frequent in malignant cases (20.0% vs. 8.6%; *p* = 0.025). Tumor marker analysis revealed significantly higher rates of elevated AFP, CA-125, and β-HCG in malignant tumors (All *p* < 0.001). CEA was undetectable in all tested cases, and no significant difference in estradiol levels was found between groups (*p* = 0.063). Ultrasound measurements demonstrated larger maximum diameters in malignant masses (105.4 ± 43.7 mm vs. 67.7 ± 45.5 mm; *p* < 0.001). Benign lesions predominantly exhibited < 20% solid component proportion (77.7%), whereas malignant lesions showed progressively higher solid component proportion (*p* < 0.001 for trend).

**Table 2:** Comparison of clinical characteristics, tumor markers, and ultrasound features between malignant and benign ovarian masses

	Benign	Malignant	*P*
No. of Patients	269	40	
No. of Cases	273	40	
Age(years), mean ± SD	8.8 ± 3.8	7.4 ± 2.9	0.01
Laterality, Right-sided, n (%)	Right: 151 (55.3%)	Right: 16(40.0%)	0.07
Chief complaint, n (%)			
Pain	146 (54.3%)	21 (52.5%)	0.834
Mass	12 (4.5%)	4 (10.0%)	0.275
Precocious puberty	23 (8.6%)	8 (20.0%)	0.025
Incidental finding	88 (32.7%)	7 (17.5%)	0.052
Tumor markers, n/N(%) ^a^			
AFP	1/124 (0.8%)	19/23 (82.6%)	<0.001
CEA	0/124 (0%)	0/23 (0%)	
CA−125	12/102 (11.8%)	14/18 (77.8%)	<0.001
Estradiol	8/43 (18.6%)	6/11 (54.6%)	0.063
* β*-HCG	0/85 (0%)	6/15 (40.0%)	<0.001
Mass size by Ultrasound(mm), mean ± SD	67.7 ± 45.5	105.4 ± 43.7	<0.001
Solid component proportion (Ultrasound imaging), n (%)			
<20%	212 (77.7%)	6 (15.0%)	<0.001
20%−40%	42 (15.4%)	8 (20.0%)	0.457
40%−60%	8 (2.9%)	11 (27.5%)	<0.001
60%−80%	9 (3.3%)	4 (10.0%)	0.119
>80%	2 (0.7%)	11 (27.5%)	<0.001

Fisher’s exact test was used for groups with expected cell counts < 5

Tumor marker elevation was defined as serum levels exceeding institutional reference ranges

^a^ N indicates number of patients tested

**Fig. 2:**
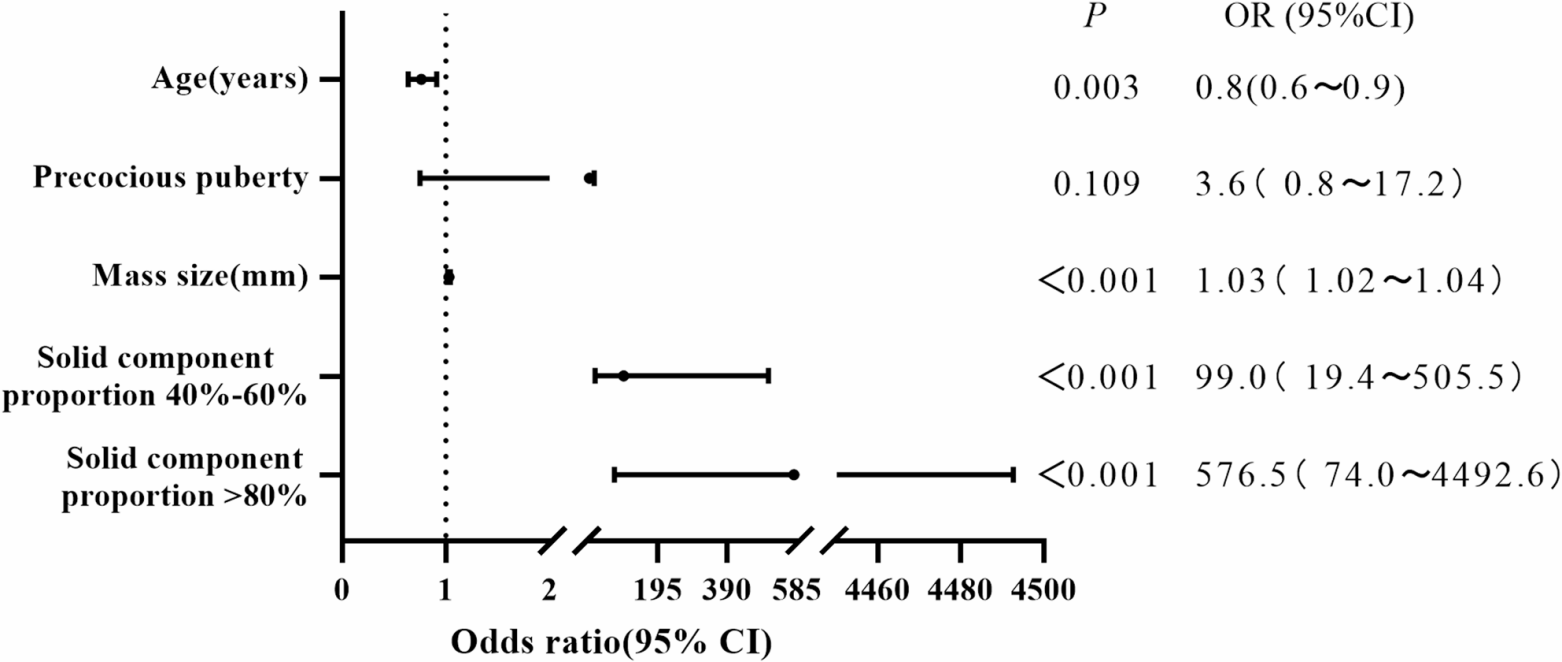
Forest plot of multivariate logistic regression analysis for predictors of malignancy

Binary logistic regression analysis was performed using age(years), precocious puberty, mass size(mm), and solid component proportion (Categorized as 40–60% and > 80% with < 20% as reference, Fig. 2). The results identified age, mass size, solid component proportion 40–60% and > 80% as key predictors of malignancy. Age demonstrated an inverse association with malignancy (OR = 0.8, 95% CI: 0.6–0.9; *p* = 0.003). Increased mass size was significantly associated with a higher risk of malignancy (OR = 1.03 per mm, 95% CI: 1.02–1.04; *p* < 0.001). Both solid component proportion 40–60% (OR = 99.0, 95% CI: 19.4-505.5; *p* < 0.001) and > 80% (OR = 576.5, 95% CI: 74.0-4492.6; *p* < 0.001) were the strongest predictors of malignant ovarian masses. Although precocious puberty did not reach statistical significance (*p* = 0.109), it showed an increased risk of malignancy (OR = 3.6, 95% CI: 0.8–17.2).

**Fig. 3:**
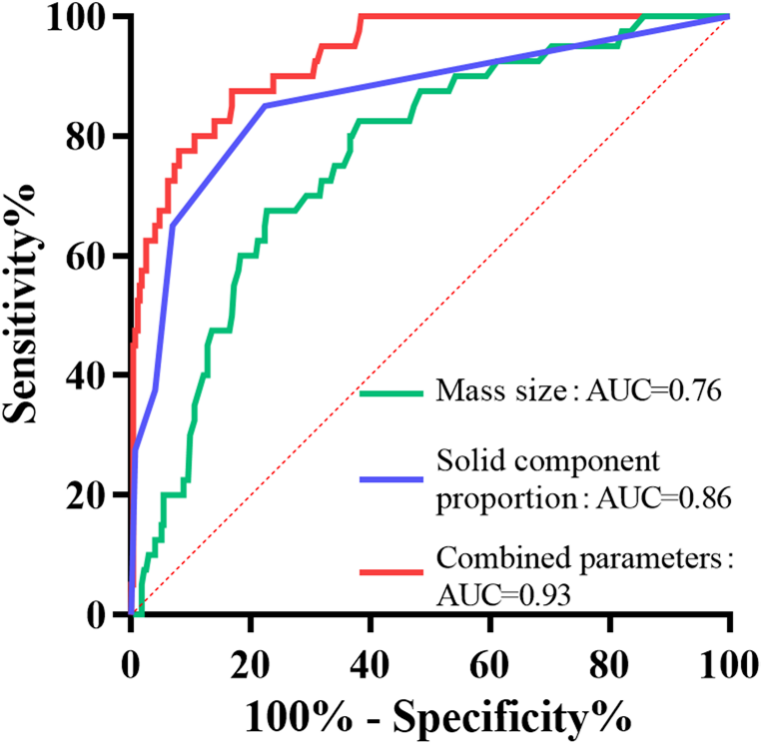
ROC curves for malignancy prediction

ROC curve analysis was performed to evaluate the diagnostic performance of ultrasound parameters-mass size, solid component proportion, and their combination-in differentiating malignant from benign ovarian masses (Fig. 3; Table 3). The solid component proportion demonstrated significantly higher diagnostic efficacy (AUC = 0.86; *p* < 0.001) compared to mass size alone (AUC = 0.76; *p* < 0.001). However, the combined model achieved excellent predictive power (AUC = 0.93; *p* < 0.001), substantially outperforming either single parameter.

**Table 3:** Diagnostic performance for differentiating malignant from benign pediatric ovarian masses using ROC curve analysis

Parameter	AUC	*p*	Cut-off value	Sensitivity(%)	Specifici(%)
Mass size	0.76	<0.001	85.5 (mm)	67.5	77.3
Solid component	0.86	<0.001	>30%	85.0	77.7
Combined model^a^	0.93	<0.001	0.097	87.5	83.2

^a^ The combined model refers to the predicted probability derived from the multivariate logistic regression model including mass size and solid component proportion

To establish reliable discriminators for reducing unnecessary surgery, we analyzed 53 epithelial tumors and 35 follicular cysts (Table 4). Patients with epithelial tumors were significantly older than those with follicular cysts (median age: 12 years [IQR 10–13] vs. 8 years [IQR 1–12]; *p* < 0.001). Laterality analysis revealed a distinct distribution pattern, with follicular cysts exhibiting a predilection for the right ovary (74.3% vs. 52.8% in epithelial tumors; *p* = 0.043). Precocious puberty was more prevalent in the follicular cyst group (37.1% vs. 13.2%; *p* = 0.009). Ultrasound measurements revealed larger maximum diameters in epithelial tumors (78 mm [IQR 54–107] vs. 50 mm [IQR 44–59]; *p* < 0.001). While both types predominantly presented as purely cystic lesions (Epithelial: 66.0%, Follicular: 100%), septations (28.3% of epithelial cysts) and psammomatous calcifications (5.7%) were exclusively observed in epithelial tumors.

**Table 4:** Comparison of characteristics between epithelial tumors and follicular cysts in pediatric ovarian masses

	Epithelial	Follicular cyst	*P*
No. of Patients	53	35	
No. of Cases	53	35	
Age(years), IQR	12 (10,13)	8 (1,12)	<0.001
Laterality, Right-sided, n (%)	Right:28 (52.8%)	Right:26 (74.3%)	0.043
Chief complaint, n (%)			
Pain	28 (52.8%)	13 (37.1%)	0.149
Mass	5 (9.4%)	3 (8.6%)	1.000
Precocious puberty	7 (13.2%)	13 (37.1%)	0.009
Incidental finding	13 (24.5%)	6 (17.1%)	0.410
Mass size(mm), IQR	78 (54,107)	50 (44,59)	<0.001
Imaging characteristic (Ultrasound), n(%)			
Purely cystic	35 (66.0%)	35 (100%)	<0.001
Presence with calcification	3 (5.7)	0 (0%)	0.273
Presence with septations	15 (28.3%)	0 (0%)	<0.001

*Abbreviation: IQR* Interquartile range

Fisher’s exact test was used for groups with expected cell counts < 5

## Discussion

This study establishes a malignancy prediction model for pediatric ovarian masses based on a large Chinese cohort (*n* = 344). Key discoveries include: ① GCTs dominate the pathological spectrum (72.7%), with mature teratoma comprising 86.6%; ② Solid component proportion > 80% emerged as the strongest malignancy predictor (OR = 576.5, *p* < 0.001); ③ The combined model (Mass size + Solid component proportion) significantly improved diagnostic accuracy (AUC = 0.932 vs. 0.86 for solid component proportion alone); ④ For follicular cyst discrimination, septations (28.3%) and calcifications (5.7%) were specific to epithelial tumors (*p* < 0.001). These findings address the critical evidence gap in Chinese pediatric ovarian oncology.

This study confirms that GCTs constituted the predominant pathological type of ovarian masses in Chinese children, accounting for 72.7% (253/348) of cases. Within GCTs, mature teratoma was the most prevalent subtype (86.6%, 219/253), aligning closely with epidemiological data from Western populations [12]. Epithelial tumors represented the second most common category (15.2%, 53/348). Notably, epithelial tumors demonstrated a striking age-dependent prevalence, with 92.5% (49/53) occurring in the 9-16-year age group. This pattern may be attributed to gonadal maturation and dynamic hormonal changes characteristic of this developmental stage [13, 14]. The overall malignancy rate in our surgical cohort was 11.5% (40/348). Among malignant lesions, GCTs were again predominant (85.0%, 34/40), with immature teratoma being the most frequent malignant subtype (55.0% of malignant GCTs, 22/40). This distribution underscores the fundamental pathological divergence between pediatric and adult ovarian masses, where epithelial carcinomas predominate [15]. However, pediatric-specific prediction models for ovarian malignancy are relatively scarce, and clinical focus remains primarily oriented towards adult malignancies [5]. Consequently, developing efficient, pediatric-specific prediction models for ovarian malignancy, such as the one validated herein, is of paramount clinical significance.

Although the initial clinical presentations of ovarian masses are diverse, abdominal pain represents the most common symptom in both benign and malignant cases. This is largely attributable to the frequent use of ultrasound as the primary screening modality for pediatric abdominal emergencies, leading to the incidental detection of many masses during abdominal scanning [16]. In our view, consequently, abdominal pain alone cannot serve as a reliable discriminator for tumor malignancy. However, precocious puberty demonstrates a statistically significant difference between benign and malignant tumors. This association is likely attributable to the hormone-secreting properties of specific malignancies, particularly sex cord-stromal tumors such as juvenile granulosa cell tumors [17].

Regarding tumor markers, although CA125 levels demonstrated a statistically significant difference between benign and malignant groups (*p* < 0.001), its sensitivity for malignancy was merely 53.8% (14/26). Our data demonstrated a weak correlation of CA125 with malignancy in children, consistent with prior criticisms of adult models like ROMA [5]. In the present study, we incorporated AFP and β-HCG, markers highly relevant to pediatric germ cell malignancies. AFP exhibited the highest sensitivity (95.0%, 19/20) for identifying malignant germ cell tumors. This exceptional performance stems from the persistent and excessive production and secretion of AFP by yolk sac tumor elements, a common component within malignant pediatric germ cell tumors [3]. Similarly, elevated β-HCG levels in children and adolescents (Excluding pregnancy) strongly suggest the presence of trophoblastic components, most frequently encountered in mixed germ cell tumors and immature teratomas [18]. By formally integrating AFP and β-HCG with key imaging parameters, our work translates established biomarker knowledge into a practical, multi-parameter risk assessment tool for the pediatric population [8, 10, 19]. Critically, postoperative monitoring reveals that declining AFP and β-HCG levels serve as favorable prognostic indicators, whereas elevated or rising levels may indicate tumor recurrence [20].

The most potent predictor in our model was a solid component proportion exceeding 80% (OR = 576.5). This finding extends beyond prior studies that often used a binary “presence of solid tissue” as a risk factor [21, 22]. By implementing a five-tiered grading system, we demonstrated a dramatic, quantifiable increase in malignancy risk with increasing solidity. Notably, we identified a solid component proportion > 40% as a critical, child-specific risk threshold, with the 40–60% category already conferring a high odds ratio (OR = 99.0). This stands in contrast to the ADNEX model, which uses a > 10% solid component threshold derived from adult epidemiology, a criterion that would likely overestimate malignancy risk in children due to the high prevalence of benign mature teratomas containing solid elements [23]. Our multiparameter model, combining this refined solid component metric with maximum tumor diameter, achieved excellent diagnostic accuracy (AUC = 0.932), which compares favorably with previously reported models in pediatric literature [7, 18].

Although epithelial tumors and follicular cysts share similar sonographic features, their clinical management differs substantially: epithelial tumors typically require surgical resection, whereas follicular cysts are functional entities that often resolve spontaneously [24]. Follicular cysts arise from immature hypothalamic-pituitary-gonadal (HPG) axis development in young children (< 8 years), rendering them susceptible to external stimuli (e.g., Obesity, Stress) that trigger excessive follicular proliferation without ovulation, ultimately leading to cyst formation [25]. In contrast, epithelial tumors originate from heightened proliferative activity of ovarian surface epithelium during gonadal maturation in adolescence, which increases cumulative genetic mutations [26]. Consequently, follicular cysts manifest at significantly younger ages than epithelial tumors. A statistical difference in laterality was observed (*p* = 0.043). However, we do not consider this to be clinically meaningful. The finding is likely a spurious association attributable to the small sample size in this subset of patients rather than a reflection of true underlying pathophysiology. Unlike epithelial tumors, follicular cysts feature granulosa cells that persistently secrete estradiol, resulting in prominent pseudoprecocious puberty [25]. Due to confinement by the ovarian cortex, follicular cysts undergo spontaneous rupture or absorption upon reaching critical dimensions, whereas epithelial tumors possess inherent proliferative capacity and sustained secretory function—enabling continuous growth unless detected due to acute complications like torsion or rupture [27–29]. Therefore, this fundamental difference in biological behavior explains why epithelial tumors are typically discovered at significantly larger sizes compared to follicular cysts. mucinous cystadenomas, the calcifications might be related to a secretory phenomenon, while true septations form through epithelial cell projections into the lumen supported by fibrovascular cores [30, 31]. Although the aforementioned features are established in the diagnosis of cystadenomas, these characteristics have not been formally incorporated into pediatric-specific risk models as key discriminators for excluding follicular cysts. The mechanistic framework elucidated herein—anchored in developmental endocrinology (HPG axis immaturity) and tumor biology (epithelial mutational accumulation)—validates our discriminative criteria (Age < 8 years, Precocious puberty, Absence of septations/calcifications), enabling a reduction in unnecessary surgeries for physiological cysts while ensuring timely intervention for true neoplasms.

Derived from multivariate analysis, our novel risk stratification framework incorporates an ultrasound-first classification protocol. Purely cystic masses (Type A): For purely cystic masses, discrimination between follicular cysts and neoplastic lesions must integrate age, size, precocious puberty manifestations, and the absence/presence of septations or calcifications—critical to avoid unnecessary resections of physiological entities. Solid-containing masses (Type B): ① Low-risk: Solid component proportion < 20% + Maximum diameter < 85.5 mm + Absence of elevated AFP, CA125, or β-HCG; ② Intermediate-risk: Solid component proportion 20–40% ± Maximum diameter ≥ 85.5 mm + Absence of elevated AFP, CA125, or β-HCG; ③ High-risk: Solid component proportion > 40% + Maximum diameter ≥ 85.5 mm ± Presence of elevated AFP, CA125, or β-HCG. This system synergistically integrates quantitative sonographic parameters (Solid component proportion, Mass size) and serum biomarkers (AFP, β-HCG, CA125), significantly enhancing diagnostic efficacy for pediatric malignancies and providing critical guidance for surgical decision-making.

Several limitations also warrant consideration. First, the potential diagnostic significance of the ovarian crescent sign was not systematically evaluated in our cohort [32]. Second, the absence of borderline epithelial tumors in our series (0/53 epithelial cases) contrasts with reported pediatric incidences of around 15% [22]. This is a notable limitation because borderline tumors may present as large, predominantly cystic masses with negative tumor markers—a profile that would be classified as low-risk by our model, suggesting possible selection bias or center-specific pathological classification practices. Most critically, the extraordinarily wide confidence intervals accompanying high odds ratios (e.g., OR = 576.5, 95% CI: 73.97-4,492.60 for solid proportion > 80%) reflect instability in risk estimation attributable to the limited number of malignant cases (*n* = 40). Nevertheless, these evidence-based advances address critical gaps and represent a step toward more accurate risk stratification of pediatric ovarian masses.

## Conclusion

This study successfully developed and validated a pediatric-specific prediction model for sonographic malignant from benign ovarian masses in Chinese children, integrating sonographic features with clinical and laboratory data. Our findings underscore the predominance of GCTs in this population and establish solid component proportion > 40% as a quantitative imaging marker for malignancy risk stratification. The combined model, incorporating mass size and solid component proportion, demonstrated superior diagnostic accuracy (AUC = 0.932), significantly outperforming individual parameters. Furthermore, in addition to patient age and mass size, the identification of septations and calcifications as features exclusive to epithelial tumors provides a reliable means to differentiate them from physiological follicular cysts, thereby reducing unnecessary surgeries. This model, which provides a practical, evidence-based tool for preoperative decision-making, helps ensure timely intervention for high-risk cases while preserving ovarian function in low-risk patients. Its design is grounded in the distinct pathophysiology of pediatric ovarian masses.

## Data Availability

The datasets generated or analyzed during the study are available from the corresponding author on reasonable request.

## Abbreviations

GCTs: Germ cell tumors
ROC: Receiver operating characteristic
AFP: Alpha fetoprotein
CEA: Carcinoembryonic antigen
AUC: Area Under the Curve
β-HCG: β-human chorionic gonadotrophin
CA125: Carbohydrate antigen 125
HE4: Human epididymis protein 4
OR: Odds ratio
CI: Confidence intervals
ROMA: The Risk of Ovarian Malignancy Algorithm
ADNEX: Assessment of Different NEoplasias in the adneXa
HPG: Hypothalamic-pituitary-gonadal
IQR: Interquartile range

## References

[1] El Helali A, Kwok GST, Tse KY. Adjuvant and post-surgical treatment in non-epithelial ovarian cancer. Best Pract Res Clin Obstet Gynaecol. 2022;78:74–85. 10.1016/j.bpobgyn.2021.06.001. 34493450 10.1016/j.bpobgyn.2021.06.001

[2] Bašković M, Habek D, Zaninović L, Milas I, Pogorelić Z. The Evaluation, Diagnosis, and management of ovarian Cysts, Masses, and their complications in Fetuses, Infants, Children, and adolescents. Healthc (Basel). 2025;13(7):775. 10.3390/healthcare13070775. Published 2025 Mar 31. 10.3390/healthcare13070775 PMC11988711 40218072

[3] De Maria F, Amant F, Chiappa V, et al. Malignant germ cells tumor of the ovary. J Gynecol Oncol. 2025;36(3):e108. 10.3802/jgo.2025.36.e108. 40275685 10.3802/jgo.2025.36.e108 PMC12099048

[4] Guo H, Chen H, Wang W, Chen L, Clinicopathological, Features. Prognostic Factors, survival Trends, and treatment of malignant ovarian germ cell tumors: A SEER database analysis. Oncol Res Treat. 2021;44(4):145–53. 10.1159/000509189. 33706324 10.1159/000509189

[5] Molina R, Escudero JM, Augé JM, et al. HE4 a novel tumour marker for ovarian cancer: comparison with CA 125 and ROMA algorithm in patients with gynaecological diseases. Tumour Biol. 2011;32(6):1087–95. 10.1007/s13277-011-0204-3. 21863264 10.1007/s13277-011-0204-3 PMC3195682

[6] Moore RG, McMeekin DS, Brown AK, et al. A novel multiple marker bioassay utilizing HE4 and CA125 for the prediction of ovarian cancer in patients with a pelvic mass. Gynecol Oncol. 2009;112(1):40–6. 10.1016/j.ygyno.2008.08.031. 18851871 10.1016/j.ygyno.2008.08.031 PMC3594094

[7] Papic JC, Finnell SM, Slaven JE, Billmire DF, Rescorla FJ, Leys CM. Predictors of ovarian malignancy in children: overcoming clinical barriers of ovarian preservation. J Pediatr Surg. 2014;49(1):144–8. 10.1016/j.jpedsurg.2013.09.068. 24439599 10.1016/j.jpedsurg.2013.09.068

[8] Madenci AL, Levine BS, Laufer MR, et al. Preoperative risk stratification of children with ovarian tumors. J Pediatr Surg. 2016;51(9):1507–12. 10.1016/j.jpedsurg.2016.05.004. 27289417 10.1016/j.jpedsurg.2016.05.004

[9] Oosterhuis JW, Looijenga LHJ. Human germ cell tumours from a developmental perspective. Nat Rev Cancer. 2019;19(9):522–37. 10.1038/s41568-019-0178-9. 31413324 10.1038/s41568-019-0178-9

[10] Billmire D, Dicken B, Rescorla F, et al. Imaging appearance of nongerminoma pediatric ovarian germ cell tumors does not discriminate benign from malignant histology. J Pediatr Adolesc Gynecol. 2021;34(3):383–6. 10.1016/j.jpag.2020.11.014. 33316416 10.1016/j.jpag.2020.11.014 PMC8096645

[11] Mentessidou A, Jackson C. Approaches to the diagnosis and management of paediatric ovarian tumours and oncological outcomes in a Single-centre Study. Evidence in support of IPSO reccomendations. J Pediatr Surg. 2025;60(2):162009. 10.1016/j.jpedsurg.2024.162009. 39467420 10.1016/j.jpedsurg.2024.162009

[12] Hermans AJ, Kluivers KB, Janssen LM, et al. Adnexal masses in children, adolescents and women of reproductive age in the netherlands: A nationwide population-based cohort study. Gynecol Oncol. 2016;143(1):93–7. 10.1016/j.ygyno.2016.07.096. 27421754 10.1016/j.ygyno.2016.07.096

[13] Tsai JY, Saigo PE, Brown C, La Quaglia MP. Diagnosis, pathology, staging, treatment, and outcome of epithelial ovarian neoplasia in patients age < 21 years. Cancer. 2001;91(11):2065–70. 10.1002/1097-0142(20010601)91:11%3C2065::aid-cncr1233%3E3.0.co;2-r. 11391586 10.1002/1097-0142(20010601)91:11<2065::aid-cncr1233>3.0.co;2-r

[14] Virgone C, Alaggio R, Dall’Igna P, et al. Epithelial tumors of the ovary in children and teenagers: A prospective study from the Italian TREP project. J Pediatr Adolesc Gynecol. 2015;28(6):441–6. 10.1016/j.jpag.2014.12.010. 26220350 10.1016/j.jpag.2014.12.010

[15] Zhang M, Jiang W, Li G, Xu C. Ovarian masses in children and adolescents - an analysis of 521 clinical cases. J Pediatr Adolesc Gynecol. 2014;27(3):e73–7. 10.1016/j.jpag.2013.07.007. 24157281 10.1016/j.jpag.2013.07.007

[16] Nicola R, Dogra V. Ultrasound: the triage tool in the emergency department: using ultrasound first. Br J Radiol. 2016;89(1061):20150790. 10.1259/bjr.20150790. 26568440 10.1259/bjr.20150790 PMC4985450

[17] Elsherif S, Bourne M, Soule E, Lall C, Bhosale P. Multimodality imaging and genomics of granulosa cell tumors. Abdom Radiol (NY). 2020;45(3):812–27. 10.1007/s00261-019-02172-3. 31410505 10.1007/s00261-019-02172-3

[18] Göbel U, Schneider DT, Calaminus G, Haas RJ, Schmidt P, Harms D. Germ-cell tumors in childhood and adolescence. GPOH MAKEI and the MAHO study groups. Ann Oncol. 2000;11(3):263–71. 10.1023/a:1008360523160. 10811491 10.1023/a:1008360523160

[19] Stankovic ZB, Djukic MK, Savic D, et al. Pre-operative differentiation of pediatric ovarian tumors: morphological scoring system and tumor markers. J Pediatr Endocrinol Metab. 2006;19(10):1231–8. 10.1515/jpem.2006.19.10.1231. 17172084 10.1515/jpem.2006.19.10.1231

[20] Gică N, Peltecu G, Chirculescu R, et al. Ovarian germ cell tumors: pictorial essay. Diagnostics (Basel). 2022;12(9):2050. 10.3390/diagnostics12092050. Published 2022 Aug 24. 36140449 10.3390/diagnostics12092050 PMC9498179

[21] Cui L, Xu H, Zhang Y. Diagnostic accuracies of the ultrasound and magnetic resonance imaging ADNEX scoring systems for ovarian adnexal mass: systematic review and Meta-Analysis. Acad Radiol. 2022;29(6):897–908. 10.1016/j.acra.2021.05.029. 34217614 10.1016/j.acra.2021.05.029

[22] Oltmann SC, Garcia N, Barber R, Huang R, Hicks B, Fischer A. Can we preoperatively risk stratify ovarian masses for malignancy? J Pediatr Surg. 2010;45(1):130–4. 10.1016/j.jpedsurg.2009.10.022. 20105592 10.1016/j.jpedsurg.2009.10.022

[23] Lala SV, Strubel N. Ovarian neoplasms of childhood. Pediatr Radiol. 2019;49(11):1463–75. 10.1007/s00247-019-04456-8. 31620847 10.1007/s00247-019-04456-8

[24] Sayasneh A, Ekechi C, Ferrara L, et al. The characteristic ultrasound features of specific types of ovarian pathology (review). Int J Oncol. 2015;46(2):445–58. 10.3892/ijo.2014.2764. 25406094 10.3892/ijo.2014.2764 PMC4277251

[25] Arisaka O, Shimura N, Nakayama Y, et al. Ovarian cysts in precocious puberty. Clin Pediatr (Phila). 1989;28(1):44–7. 10.1177/000992288902800111. 2910632 10.1177/000992288902800111

[26] Pereira M, Matuszewska K, Jamieson C, Petrik J. Characterizing endocrine Status, tumor hypoxia and immunogenicity for therapy success in epithelial ovarian cancer. Front Endocrinol (Lausanne). 2021;12:772349. 10.3389/fendo.2021.772349. Published 2021 Nov 17. 34867818 10.3389/fendo.2021.772349 PMC8635771

[27] Bhuyan G, Arora R, Ahluwalia C, Sharma P. Epithelial-mesenchymal transition in serous and mucinous epithelial tumors of the ovary. J Cancer Res Ther. 2019;15(6):1309–15. 10.4103/jcrt.JCRT_35_18. 31898665 10.4103/jcrt.JCRT_35_18

[28] Sutton CL, McKinney CD, Jones JE, Gay SB. Ovarian masses revisited: radiologic and pathologic correlation. Radiographics. 1992;12(5):853–77. 10.1148/radiographics.12.5.1529129. 1529129 10.1148/radiographics.12.5.1529129

[29] Stewart CJR, Harding S. Stromal endocrine cell micronests associated with an ovarian mucinous cystadenoma: endocrine cell preservation (Pseudohyperplasia) potentially mimicking stromal sex cord proliferation or tumor microinvasion. Int J Gynecol Pathol. 2021;40(1):56–9. 10.1097/PGP.0000000000000646. 31688244 10.1097/PGP.0000000000000646

[30] Silva EG, Deavers MT, Parlow AF, Gershenson DM, Malpica A. Calcifications in ovary and endometrium and their neoplasms. Mod Pathol. 2003;16(3):219–22. 10.1097/01.MP.0000057236.96797.07. 12640101 10.1097/01.MP.0000057236.96797.07

[31] Katzenstein AL, Mazur MT, Morgan TE, Kao MS. Proliferative serous tumors of the ovary. Histologic features and prognosis. Am J Surg Pathol. 1978;2(4):339–55. 10.1097/00000478-197812000-00001. 736209 10.1097/00000478-197812000-00001

[32] Stankovic Z. Ovarian cysts and tumors in adolescents. Obstet Gynecol Clin North Am. 2024;51(4):695–710. 10.1016/j.ogc.2024.08.006. 39510739 10.1016/j.ogc.2024.08.006

